# New Trigger for Stroke-like Episode in Sturge–Weber Syndrome: A Case Report

**DOI:** 10.3390/children12050589

**Published:** 2025-04-30

**Authors:** Emiliano Altavilla, Andrea De Giacomo, Anna Maria Greco, Fernanda Tramacere, Marilena Quarta, Daniela Puscio, Massimo Corsalini, Sara Pistilli, Dario Sardella, Flavia Indrio

**Affiliations:** 1 Department of Sperimental Medicine Pediatric Section, University of Salento Hospital “Vito Fazzi”, 73100 Lecce, Italy; eminoc@libero.it (E.A.); annamariagreco1968@libero.it (A.M.G.); ftramacere@libero.it (F.T.); marilena.quarta@libero.it (M.Q.); flaviaindrio1@gmail.com (F.I.); 2 Department of Child Neuropsychiatry, Policlinico of Bari University Hospital, 70124 Bari, Italy; s.pistilli97@gmail.com (S.P.); dario.sardella@gmail.com (D.S.); 3 Department of Anesthesia and Resuscitation, University of Salento Hospital “Vito Fazzi”, 73100 Lecce, Italy; danipuscio@gmail.com; 4 Department of Interdisciplinary Medicine, University of Bari “Aldo Moro”, 70100 Bari, Italy; massimo.corsalini@uniba.it

**Keywords:** Sturge–Weber syndrome, stroke-like event, deep sedation

## Abstract

**Background**. Sturge–Weber syndrome (SWS) is a rare non-hereditary neurovascular disorder characterized by capillary–venous malformations on the face, ocular vascular anomalies, and leptomeningeal capillary–venous malformations. Patients with SWS often experience cerebral perfusion impairment, increasing their risk for stroke-like episodes, seizures, and motor and cognitive impairments. **Methods**. We report the case of a 2-year-old boy diagnosed with SWS who developed a stroke-like episode following dye laser therapy under deep sedation. **Results**. Despite initial diagnostic challenges and persistent seizures, appropriate management led to full neurological recovery. **Conclusions**. This case highlights the importance of considering stroke-like episodes in children with SWS after stressful events such as medical procedures.

## 1. Introduction

### 1.1. History

The association between facial angioma and buphthalmos was described by Schirmer in 1860; however, the relationship between the two phenomena had not been identified [1].

Sturge–Weber syndrome (SWS) was firstly described in the 1800s, in a 6-year-old girl with a very extensive ‘mother’s mark’, which is known today as ‘port-wine birthmarks’ (PWBs), on the right side of the head and face. Although after the birth she enjoyed good health, starting from the sixth month she began to have multiple daily attacks, which, over time, had occurred with the loss of consciousness and side effects upon awakening (e.g., the children felt weakness on her left side and had difficulty walking) [2].

Nowadays, Sturge–Weber syndrome is considered as one of the more common neurocutaneous disorders, with an incidence of 1:20,000 to 1:50,000 live births. It is the third most common neurocutaneous disorder after Neurofibromatosis and Tuberous Sclerosis [3].

### 1.2. Genetics

Sturge–Weber syndrome is commonly caused by somatic, mosaic variants of the GNAQ gene, which encodes the G-protein subunit α_q_, which is associated with a GPCR (G-Coupled Cell Receptor). GPCRs are associated with Gαq, which modulates cellular processes by influencing protein activity and phosphorylation, essential mechanisms for cellular proliferation and differentiation. The p.R183Q mutation results in the constitutive activation of Gαq and in the hyperactivation of downstream pathways in endothelial cells with the overproduction of angiopoietin-II, resulting in capillary malformations [4]. The R183Q mutation is found in close to 90% of patients with SWS. Other genes such as GNA11 (paralogue) variants have been described, with subtle phenotypic particularities such as syndromic capillary malformations [5]. A genetic mosaic means that patients have cells with non-pathological copies of the gene interspersed with cells containing abnormal copies; patients can also have variability in the severity of symptoms, in part depending on the extent of their involvement [6].

### 1.3. Physiopathology

Histological features are represented by enlarged blood vessels, which tend to lack mural cell coverage if compared to healthy vessels; for these reasons, they are plausible phenomena of active remodeling, altered permeability, and stasis.

A recent study by Nasim et al. demonstrated the importance of the perivascular microenvironment in the pathogenesis of Sturge–Weber syndrome (SWS). In particular, it explored the role of macrophages expressing the marker set MRC1+/CD163+/CD68+/LYVE1+. An analysis of brain samples of SWS patients revealed an environment that is densely populated by macrophages. Furthermore, endothelial cells expressing the GNAQ R183Q mutation were found to facilitate macrophage transmigration across the endothelium of capillary malformations. According to these authors, the diapedesis process could be facilitated by two factors: (i) the slowing of blood flow caused by the capillary malformation and (ii) the possible role of ICAM1, an adhesion molecule, in combination with the mechanical activation of the PIEZO1 channel [7].

Another perspective on the pathogenesis of the disease is provided by a recent study investigating the role of EDN3+ meningeal fibroblasts. EDN3 is a cardiovascular peptide involved in both physiological and pathological vascularization. The expression of this marker is associated with the presence of fibroblasts during disease progression. According to this hypothesis, the peptide may stimulate the non-canonical WNT signaling pathway, leading to dysregulated angiogenesis downstream of the process. This makes EDN3 a potential therapeutic target worthy of further exploration [8].

The presence of neurovascular calcifications is a typical finding in Sturge–Weber syndrome. The presence of deposits increases the risk of the future development of seizures and stroke-like episodes, its pathogenesis may depend on altered calcium homeostasis. Cellular calcium is an important second messenger of numerous cellular processes, and its concentration in blood flow is directly regulated by the action of parathormone (PTH) and 1,25-dihydroxicholecalciferol, and indirectly by cellular species like fibroblast FGF23+. Authors have demonstrated the anomaly of calcium homeostasis processes within the context of ionized hypocalcemia with the normal action of PTH, and have hypothesized a multifactorial outcome in which the frequent administration of antiepileptic drugs such as levetiracetam and oxcarbamazepin must be considered [9].

### 1.4. Neuroimaging

MRI criteria for the assessment of neuroradiological features in Sturge–Weber syndrome (SWS) include the following: the presence of leptomeningeal capillary malformations, the extent of lobar cortical atrophy, abnormal white matter signals, cortical calcifications, and choroid plexus abnormalities [10]. Specific protocols should be developed to screen patients with SWS type III, as these individuals may remain undiagnosed until undergoing neuroimaging for acute migraine, stroke-like events, or other unrelated reasons [11]

Contrast-enhanced MRI performed before 3 months of age can suggest a vascular anomaly and facilitate early diagnosis. Detection in a presymptomatic stage may result in improved long-term outcomes [12].

Some authors recommend early baseline contrast-enhanced MRI to avoid diagnostic uncertainty and to allow timely referral for specialist evaluation [13]. In very young patients, indirect MRI signs may be observed, such as ipsilateral choroid plexus enlargement and the signal inversion of the white matter (suggestive of white matter abnormalities or cerebral atrophy) [14]. These findings are supported by a recent study, which identified dilated venous channels as potential early compensatory biomarkers in patients with SWS. The venous channels evaluated included the subarachnoid varicose network, transmedullary veins, subependymal veins, and the choroid plexus. These phenomena precede cortical atrophy, calcifications, and the formation of leptomeningeal angiomas [15].

Considering the risks and benefits of sedation in newborns, Sabeti et al. suggest that children with a high-risk port-wine stain (PWS) but no seizures do not require early imaging unless presymptomatic treatment is being considered [16].

## 2. Clinical Manifestations

Clinical manifestations of SWS include facial port-wine stains (PWSs), leptomeningeal angiomas, glaucoma, choroidal hemangioma, anatomic malformations, and oral malformations; neurological complications such as migraine, seizures, stroke-like episodes, and cognitive delays are also common [17].

However, as we can read in the literature, long-term outcomes near to a normal life can be achieved. For this reason, we report the case of the pregnancy management of a female patient with SWS [18].

A disease with such multisystemic disorders should be managed by a multidisciplinary team. The early recognition of its manifestations can often lead to timely treatment and improved long-term outcomes [19].

### 2.1. Encephalofacial Angiomatosis

The Roach Scale classification was one of the first, and now outdated, classifications of SWS, which aimed to clinically distinguish between isolated facial involvement and associated leptomeningeal involvement. It divided *encephalofacial angiomatosis* as follows: Type I: both facial and leptomeningeal malformations, with the possibility of glaucoma (classic SWS). Type II: facial capillary malformation alone, with the possibility of glaucoma. Type III: isolated *leptomeningeal angiomatosis*, usually without glaucoma [20]. Nowadays, it is known that the syndromic association to SWS and the risk of brain and eye involvement can be stratified based on the extension and location of the facial port-wine birthmark (PWB) (Table 1).

A predictive role for decisional algorithms has been proposed to evaluate eligibility for surgical resection; however, further studies are warranted to validate their use [21].

The presence of a lower eyelid and choroidal angiomas were linked to glaucoma genesis. No association was observed with leptomeningeal angiomas, possibly due to their greater distance from the eye [22].

The gold standard treatment for PWB is pulsed dye laser (PDL) treatment, which specifically targets hemoglobin within cutaneous blood vessels. Although various laser technologies are utilized for the treatment of vascular skin lesions, PDL treatment remains the most effective and has the most well-established safety profile, particularly in pediatric patients [23].

### 2.2. Ocular Manifestations

Up to half of the patients with SWS have a variable degree of ocular abnormalities. The two most common manifestations of ocular affection are glaucoma and choroidal hemangiomas; other vascular anomalies in the eye can involve the conjunctiva, episclera, retina, and/or choroid, potentially resulting in optic atrophy and blindness [24].

#### 2.2.1. Glaucoma

The ocular parameters relevant for glaucoma diagnosis, aside from intraocular pressure (IOP), include variations in corneal diameter, axial length, and cup-to-disc ratio. Additionally, symptoms such as blepharospasm, excessive tearing, and photophobia may indicate a suspicion of glaucoma [25].

In the context of SWS, the most common eye problem is glaucoma, classically ipsilateral to facial PWB or leptomeningeal angiomatosis; it can be congenital or appear later in life. Up to 30–70% of patients with SWS develop glaucoma, and 60% of them develop it during childhood. Glaucoma in the SWS context is difficult to manage, often resistant to both medical and surgical treatments, and requires longitudinal monitoring due to its lifelong risk [26].

Recent studies demonstrate that early and late glaucoma in SWS patients need to be considered as different entities, each of which has its own pathogenetic mechanisms, clinical presentations, primary surgical choices, and outcomes [27].

A study on 34 neonates tried to stratify the risk of developing glaucoma based on the localization and extension of PWB; we report those features in Table 2 [28].

A surgical approach is usually needed if an early involvement is demonstrated, and the authors of [29] used an off-label procedure with a precision medicine approach in a 5-year-old patient with a good recovery.

Deep sclerotomy is a valid therapeutic option with a higher safety profile and fewer postoperative complications due to the gradual outflow of the aqueous humor through an intact trabeculo-descemet’s window, preventing complications [30].

#### 2.2.2. Choroidal Hemangioma

Choroidal hemangioma (CH), a benign vascular tumor, is present in 40% to 50% of patients with Sturge–Weber syndrome (SWS). Its presentation is usually diffuse, but it can also be circumscribed and is typically located ipsilateral to PWB. It results from abnormal and enlarged blood vessels and vascular channels, leading to various clinical manifestations such as reduced visual acuity, refractive errors, scotoma, or retinal detachment [31]. Due to the driver mutation, the lesions exhibit a proliferative nature, leading to the enlargement of the choroidal plexus [32]. The differential diagnosis is with amelanotic choroidal melanoma, which requires indocyanine green angiography and ultrasonography, and choroidal metastases which differs for the size, imaging, and number of localizations [33].

Considering the lesion posterior localization of the eye, choroidal hemangioma is challenging to treat and has a poor visual prognosis. The treatment process begins when the exudative and neovascular lesion’s components create visual impairment, and multimodal ophthalmological imaging is required [34].

### 2.3. Neurological Manifestations

Neurological manifestations of SWS include seizures, headache, and stroke-like episodes, and intellectual and language impairments are frequently associated [35].

#### 2.3.1. Epilepsy

The pathophysiological mechanisms underlying epilepsy in Sturge–Weber syndrome (SWS) remain incompletely understood; evidence suggests a link between cerebral hypoperfusion and epileptogenesis. Some studies propose that chronic ischemia may facilitate seizure generation, whereas others have identified a correlation between the number and extent of intracranial calcifications and both seizure severity and resistance to antiseizure medications [36].

Epilepsy is highly prevalent in Sturge–Weber syndrome (SWS), affecting approximately 70% of individuals with unilateral leptomeningeal capillary malformations and up to 90% of those with bilateral involvement. Most seizures occur within the first two years of life, with focal seizures (particularly focal motor clonic seizures) being the predominant type [37]. Among the 171 patients enrolled, 75% of those who developed seizures became symptomatic within the first year of life, 11% during the second year, and only 14% after the age of two. This observation suggests that the likelihood of developing epilepsy after the age of two in the context of Sturge–Weber syndrome (SWS) decreases progressively [38].

Due to the lack of gadolinium enhancement in the initial MRI, many very young patients experience a diagnostic delay. In these cases, the first seizures are often prolonged and unexpected, with the subsequent administration of phenytoin [13].

Only a few studies have investigated the response to antiseizure medications (ASMs) in patients with Sturge–Weber syndrome (SWS). A European study aimed to evaluate the effects of monotherapy and polytherapy with antiepileptic drugs and found that oxcarbazepine (OXC) or carbamazepine were the most commonly used ASMs in monotherapy among seizure-free patients, while levetiracetam (LEV) was the most frequently used ASM in monotherapy among patients with uncontrolled seizures [39]. This finding is coherent with the current literature [40].

A possible role of CBD has been proposed in refractory epilepsies, as in Dravet syndrome and Lennox–Gastaut syndrome [41].

Epilepsy surgery may be considered for patients, typically children, with SWS and drug-resistant seizures. Surgery is primarily reserved for cases with unilateral brain involvement, with rare exceptions. The most common surgical procedure is hemispherectomy, while posterior resection, preserving the frontal lobe, including the motor cortex, is performed in patients with an intact frontal lobe [38].

There are several hemispherectomy surgical techniques: anatomical hemispherectomy, transsylvian peri-insular hemispherectomy, and the vertical parasagittal disconnection of the hemisphere. In a review with a total of 1102 patients who underwent hemispherectomy, overall seizure freedom was 73.4% [42]. In another piece of the literature, the overall seizure freedom was even higher, reaching 86%; moreover, for anatomical hemispherectomy, this result was 100% [43].

A study has provided class IV evidence that in children with Sturge–Weber syndrome and pharmacoresistant epilepsy, surgical resection—whether focal resection, hemispherectomy, or modified hemispherectomy—leads to better cognitive outcomes. However, the scientific evidence is not sufficient to determine whether the age at time of surgery influences the outcome of focal resection [44].

The decision regarding the timing of surgical intervention should be made on a case-by-case basis. Antiseizure medications (ASMs) remain the first-line treatment for epilepsy management in SWS. Given that seizures in SWS are linked to structural brain lesions, most patients will require lifelong ASM therapy. However, epilepsy surgery may enable a subset of patients to discontinue antiepileptic drugs (AEDs) and aspirin [45]. The presurgical evaluation includes age-specific neuropsychological assessments, as well as additional investigative techniques such as PET, magnetic resonance spectroscopy (MRS), functional MRI (fMRI), MEG, SPECT, and invasive EEG.

A recent study highlighted the possible role of Vagus Nerve Stimulation (VNS) for the treatment of uncontrolled epilepsy. All patients enrolled demonstrated a reduction in seizure frequency and two of them exhibited cognitive improvements [46].

There is some evidence that in the case of a presymptomatic diagnosis, prophylactic treatment with aspirin and antiepileptic drugs may delay seizure onset [47]. Recently, a study proposed an early evaluation of all high-risk PWB patients by a Sturge–Weber specialist and an early diagnostic procedure through which anamnestic details and EEG findings can facilitate the prediction of seizure onset in 2-year-old children; further, adding an MRI without contrast can help the timing of therapy administration [48]. This seizure-free period could allow very young patients to have a more neurotypical development, with a lower risk of future cognitive impairments [49].

#### 2.3.2. Headache/Migraine

Up to 50% of patients with SWS also experience headaches, possibly related to transient poor blood flow triggering cortical spreading depression [50]. Common characteristics include triggers such as sleep deprivation or onset upon awakening. Beyond frequency and severity, approximately 40% of individuals with headaches report experiencing more than one episode per month; the majority suffer from some level of impaired functional ability during headache attacks [51].

Recent studies have considered triptans as a safe and effective choice for the acute treatment of patients with SWS. The administration of a vasoconstrictive molecule with a possible effect on leptomeningeal blood vessels, in addition to theoretical concerns, resulted in no side effects in this study [52].

Currently, there are no official guidelines on preventing headaches in SWS. In most countries, rest, hydration, NSAIDs, paracetamol, and antiemetics are commonly used as symptomatic treatments [53].

The administration of anti-seizure medications for migraine prophylaxis can improve both seizures and headache/migraine attacks [25].

#### 2.3.3. Stroke-like Episodes

One of the most devastating and concerning symptoms in patients with Sturge–Weber syndrome (SWS) are stroke-like episodes. These episodes are functionally defined as the onset of a new neurological deficit that persists for more than 24 h, with or without concurrent seizures [54]; in some cases, neurological deficits may include hemiplegic migraine and prolonged visual auras [55].

Stroke-like episodes related to SWS can be triggered by seizures, head trauma, or illness, but they may also occur without any obvious cause. Additionally, the differential diagnosis for stroke-like episodes includes conditions such as complicated migraines and Todd’s paralysis [56].

The use of aspirin is recommended for the treatment of hemiparetic stroke-like episodes, aiming to reduce ischemia associated with vascular malformations. Moreover, aspirin has been administered in combination with levetiracetam, both presymptomatically and following symptom onset [57].

In fact, some authors have suggested a prophylactic treatment with low-dose aspirin, showing promising results in the reduction in stroke-like episodes and better seizure control. This benefit for both complications may result from the prevention of thrombosis in the context of venous stasis, which improves blood flow from/to the brain and reduces seizure risk [58]. The suitability for the low-dose aspirin treatment is based on the severity and degree of brain involvement and all the symptoms the patient exhibits.

#### 2.3.4. Dentistry/Maxillofacial

Oral manifestations are present in 50% of patients [59].

The first dental assessment should be performed soon after the diagnosis of SWS has been formulated, in order to allow an early evaluation of possible mucosal vascular lesions, dental alterations in terms of size and/or the timing of tooth replacement, gingival hyperplasia, and skeletal asymmetries. If there are no lesions in the oral mucosa, follow-up should be every six months [25].

Gingival hyperplasia and angiomatous lesions of the oral cavity can be found in patients with SWS. Even if resolved through a surgical approach, these conditions may be characterized by a variable recurrence rate, depending on the patient and the procedure itself (scalpel, CO_2_ laser, neodymium-doped yttrium aluminum garnet laser, etc.) [60]. When a clinician identifies gingival hyperplasia, it may be plaque-induced, related to oral mucosal development driven by mutations, secondary to antiepileptic medications, or a combination of these factors [61].

The patients should also be encouraged and motivated to adopt a healthy lifestyle and avoid other risk factors that may exacerbate the condition, such as smoking, stress, or poor nutrition [62].

#### 2.3.5. Intellectual and Language Impairments

Cognitive outcomes were linked to the number of affected brain lobes, with bilateral brain involvement being associated with more severe intellectual disabilities and language disorders. Earlier seizure onset was correlated with intellectual and language disabilities, while active epilepsy was specifically associated with language disorders [63]. Patients with a port-wine birthmark (PWB) had higher rates of intellectual disabilities and language disorders compared to those without PWB. This difference may be due to the mutation occurring later in fetal development in individuals without a birthmark, leading to fewer affected cells. Furthermore, the absence of a facial PWB was associated with a later age of seizure onset, which is a key predictor of cognitive and neurological outcomes [64]. A systematic retrospective review on 70 patients with Sturge–Weber syndrome (SWS) by Sudersanam et al. revealed a clinical diagnosis of autism spectrum disorder (ASD) in 40% of cases. Using the Social Responsiveness Scale 2 (SRS-2), these patients demonstrated a greater degree of impairment in the Social Awareness scale compared to autistic individuals without an underlying clinical condition. Moreover, 30% of patients who did not meet the criteria for autism exhibited moderate to severe difficulties with social communication, while only one-third of patients had scores within the normal range [65].

Another aspect to be considered is the comorbidity with other neuropsychiatric conditions like suicidality. In a study conducted by Sebold et al. using the Ask Suicide Questions (ASQ) tool as screening tool in a sample of patients from 8 to 17 years old, a higher rate of suicidal intent compared to other neurological syndromes was observed [66].

#### 2.3.6. Outcome Measures

The SWS-Neurological Rating Score (SWS-NRS), or Neuroscore, is used as a longitudinal tool for the assessment of the extent of neurological impairment in patients with SWS [67]. The Neuroscore is a scale that sums up the scores of the level of visual impairment, frequency of seizures, level of hemiparesis, and cognitive functions, and considers childhood or adulthood. It is a rapid tool of assessment used in other studies to correlate EEG, MRI, and even pharmacological response with the clinical status of the patient; in the future, this could represent a measurement in the decisional process of administering a treatment or a therapy [68].

We decided to summarize the previously mentioned clinical manifestations, along with other less common ones, in Table 3 [64].

## 3. Case Presentation

The patient is a 3-year-old boy with a known diagnosis of SWS, which was established at age one; the patient had been on dual antiepileptic therapy with carbamazepine and clobazam due to a history of epilepsy. He exhibited typical SWS-associated facial port-wine stains and leptomeningeal involvement on brain imaging [69]. Informed consent was obtained from the patient’s parents.

### 3.1. Medical History

The child’s prior clinical course was marked by well-controlled seizures until he underwent dye laser therapy for facial capillary malformations under deep sedation. The patient did not stop antiepileptic medication for the procedure and the procedure was uneventful itself. For these reasons, he was discharged on the same day without complications [70].

### 3.2. Clinical Course

Day 1–3 Post-Procedure: the patient developed recurrent vomiting episodes, followed by sudden-onset right-sided hemiparesis and aphasia. His family noted an altered mental status and difficulty in speech production. He was brought to the emergency department (ED), where he exhibited fluctuating consciousness, persistent focal seizures, and worsening left-side hemiparesis [71].

### 3.3. Diagnostic Work-Up

Due to his neurological deterioration, an urgent brain MRI was ordered; however, it was delayed because of logistical challenges. A lumbar puncture was performed, revealing no abnormalities in the cerebrospinal fluid (CSF) analysis, ruling out infectious and autoimmune encephalitis. Electroencephalography (EEG) showed continuous epileptic activity over the left hemisphere, suggesting ictal activity with a potential stroke-like pathology [72].

### 3.4. Treatment and Manager

Empirical intravenous ceftriaxone was initiated due to the initial concern for central nervous system infection. Despite escalating doses of carbamazepine and clobazam, his seizures persisted, prompting a transfer to the pediatric intensive care unit (PICU). Continuous intravenous midazolam infusion (45 mg/50 mL) was administered, leading to seizure resolution within 48 h [70].

### 3.5. Recovery

**Figure 1:**
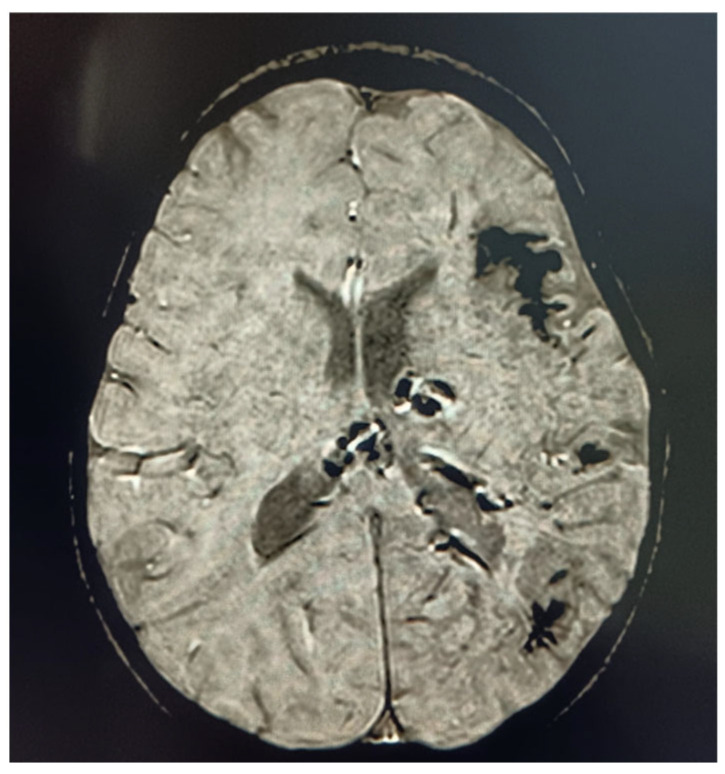
The MRI using the SWiP sequence reveals the characteristic radiological features of Sturge–Weber syndrome with multiple subcortical calcifications located in the fronto-insulo-temporal region but shows no evidence of recent ischemic areas.

The day following the resolution, a neurological examination revealed hyposthenia of the left upper and lower limbs, accompanied by paresis of the seventh cranial nerve. The child was alert, conscious, and cooperative in executing simple commands. The patient’s neurological status improved significantly, with a complete resolution of hemiparesis and restoration of speech within five days of intensive management. The next day follow-up MRI revealed no new ischemic lesions [Figure 1], confirming a reversible stroke-like episode rather than structural brain damage [27]. The DWI sequence study revealed no areas of restricted tissue water diffusivity. In light of the first presentation of a stroke-like episode, no prophylactic therapy with low-dose aspirin was initiated.

## 4. Discussion

Laser treatment for PWB typically begins in infancy, as the flat, pink birthmark responds best at this stage and is smaller in size. Early laser intervention may help prevent the later progression of the birthmark, which can include tissue hypertrophy, blebs, and complications impacting vision, airways, and swallowing [70]. Our patient is a 3-year-old boy who underwent dye lase therapy for this reason.

Stroke-like episodes in SWS can present acutely and mimic infectious, autoimmune, or ischemic processes, and can present with seizures, status epilepticus, or alone [28]. The child in our case report experienced a sudden onset and rapid escalation of symptoms. The presentation with hemiparesis is common in this type of pathology, as well as in other conditions included in the differential diagnosis of stroke-like episodes (hemorrhagic stroke, ischemic stroke, transient ischemic attack, Todd’s paralysis, etc.). Stroke-like episodes are hypothesized to result from impaired cerebral perfusion due to vascular anomalies and increased metabolic demands during stress [73]. The triggering role of sedation-related hypotension, during laser treatment, in this case emphasizes the vulnerability of SWS patients undergoing medical procedures. The previous literature reports similar events triggered by febrile illnesses, trauma, or surgical interventions [74]. The potential role of aspirin in the treatment of such transient weakness in SWS has been suggested in a study of 14 patients; a 65% decrease in stroke-like events was found after the administration of low-dose aspirin (2–3 mg/kg/d) [25]. However, there are no official guidelines for pediatricians, and our patient fully recovered after 5 days from the episode. No prophylactic therapy has been prescribed.

Epilepsy in Sturge–Weber syndrome can be challenging to treat, occurring in clusters of seizures and episodes of status epilepticus. EEG monitoring is crucial for detecting subclinical seizures, while MRI can identify cerebral ischemia or stroke-like changes [55]; in our case, the EEG showed continuous electrical activity suggestive of status epilepticus, taking priority for intervention over other considerations. The treatment involves controlling seizures and optimizing cerebral perfusion; for this reason, early seizure suppression using a continuous midazolam infusion played a crucial role in achieving a favorable outcome for this patient.

## 5. Conclusions

This case underscores the need for heightened clinical awareness of stroke-like episodes in children with SWS after medical procedures involving anesthesia or sedation. Early recognition and aggressive seizure management can result in complete neurological recovery, as demonstrated by this case. Future research should explore predictive biomarkers for identifying at-risk children and preventing stroke-like episodes in SWS [75]. This research received no external funding.

## Figures and Tables

**Table 1:** Risk stratification for the development of Sturge–Weber syndrome (SWS) based on distinct facial port-wine birthmark (PWB) phenotypes.

Distinctive Forehead PWS Phenotypes	High-Risk Facial PWS Phenotypes
Very high risk (forehead bilateral)	Hemifacial
High risk (>50% of hemi-forehead)	Forehead and upper eyelid
Low risk (localized linear)	Medial

**Table 2:** High risk for the development of glaucoma based on distinct facial port-wine birthmark (PWB) phenotypes [28].

High-Risk Patients for Development of Glaucoma Based on the Location and Extension of the Port-Wine Stain
Extensive port-wine birthmark
Port-wine birthmark involving frontal and large malar area
Lower lid involvement Right and bilateral port-wine birthmark Male gender (<4-year-old patients)

**Table 3:** Clinical manifestations in different organs to be evaluated and followed in patients with Sturge–Weber syndrome [64].

Subspeciality	Clinical Manifestations to Be Evaluated and Followed
Dermatology	Port-wine birthmark
	Port-wine birthmark elsewhere
	Overgrowth
Ophthalmology	Glaucoma
	Choroidal and Episcleral Hemangiomas Visual field defect Retinal Occlusion Ocular melanocytosis
Neurology	Seizures and Epilepsy Headache Stroke-like episodes
Orthopedic Surgery/Physiotherapy	Growth asymmetry
	Hypertrophy or hypotrophy
	Secondary scoliosis
	Functional limitations
Dentistry/Maxillofacial	Gingival and palatal angiomatosis
	Gingival hyperplasia Pre and intra-operative planning for dental surgery/procedures

## Data Availability

The data presented in this study are available on request from the corresponding author due to privacy reasons.
